# Transgenic Rescue of Spermatogenesis in Males With *Mgat1* Deleted in Germ Cells

**DOI:** 10.3389/fcell.2020.00212

**Published:** 2020-04-02

**Authors:** Barnali Biswas, Frank Batista, Ayodele Akintayo, Jennifer Aguilan, Pamela Stanley

**Affiliations:** ^1^Department of Cell Biology, Albert Einstein College of Medicine, New York, NY, United States; ^2^Laboratory for Macromolecular Analysis and Proteomics, Department of Pathology, Albert Einstein College of Medicine, New York, NY, United States

**Keywords:** spermatogenesis, MGAT1, N-glycans, fertility, transgenic rescue

## Abstract

MGAT1 and complex N-glycans are required for spermatogenesis and fertility. Conditional deletion of *Mgat1* in spermatogonia (*Mgat1* cKO) causes reduced ERK1/2 signaling and the formation of multinucleated germ cells (MNC). Here we show that glycomics analysis of N-glycans released from fixed testis sections and analyzed by MALDI imaging mass spectrometry (MALDI-IMS) revealed a loss of MGAT1 activity in all germ cells based on the accumulation of the oligomannosyl substrate of MGAT1. To determine in which type of germ cell MGAT1 is essential for spermatogenesis, we generated *Mgat1* cKO males that also expressed a *Mgat1-HA* transgene under the control of a germ cell-specific promoter – *Stra8* for spermatogonia, *Ldhc* for spermatocytes and *Prm1* for spermatids. Males expressing each *Mgat1-HA* transgene were fertile, and both males and females transmitted each transgene. When *Stra8*-*Mgat1*-HA was expressed in *Mgat1* cKO males, spermatogenesis was rescued based on the morphology of testis sections, the complement of N-glycans on basigin, lectin histochemistry, MALDI-IMS, and fertility. By contrast, neither *Ldhc-Mgat1-HA* expressed in spermatocytes, nor the *Prm1*-*Mgat1-HA* transgene expressed in spermatids rescued spermatogenesis or fertility in *Mgat1* cKO males. Therefore, MGAT1 must be expressed in spermatogonia for spermatogenesis to proceed normally.

## Introduction

MGAT1 is the glycosyltransferase that transfers GlcNAc to Man_5_GlcNAc_2_Asn to initiate complex N-glycan synthesis (reviewed in Stanley et al., 2015–2017). We previously showed that deletion of *Mgat1* in spermatogonia using *Stra8-iCre* prevents the formation of sperm, and thus causes infertility in male mice (Batista et al., 2012). The block in spermatogenesis is at the spermatid stage of germ cell differentiation. Spermatids lacking *Mgat1* form multinuclear cells (MNC) or syncytia by ∼26 days post-partum (dpp), but the number and morphology of spermatogonia, spermatocytes and Sertoli cells appear unperturbed (Biswas et al., 2018). Here we address two questions – (1) does mis-expression of *Mgat1* in spermatogonia, spermatocytes or spermatids disrupt spermatogenesis? and (2) if not, can the block in spermatogenesis in *Mgat1* cKO males be rescued by expressing a *Mgat1* transgene in spermatogonia, spermatocytes, or spermatids? The germ cell specific promoters that we used to express *Mgat1* in each germ cell type have been characterized previously – the *Stra8* promoter was used for expression in spermatogonia (Sadate-Ngatchou et al., 2008), the *Ldhc* promoter was used for expression in spermatocytes (Li et al., 1998), and the *Prm1* promoter was used for expression in spermatids (O’Gorman et al., 1997). In this paper, we show that males expressing each *Mgat1* transgene were viable and fertile. Males in which *Mgat1* had been deleted in spermatogonia (*Mgat1* cKO) and were also expressing a *Mgat1-HA* transgene in spermatocytes or spermatids exhibited the spermatogenic defects typical of *Mgat1* cKO males, and were therefore not rescued by the respective *Mgat1-HA* transgene. However, *Mgat1* cKO males expressing the Stra8-*Mgat1-HA* transgene were fertile, and thus rescued by the transgene.

## Materials and Methods

### Mice

The Transgenic Mouse Facility of the Albert Einstein College of Medicine generated the transgenic mice used in this study on a FVB/NJ background. FVB/NJ mice from Jackson Laboratories (Portland, ME, United States) were used for breeding. All mice carrying a transgene were maintained as heterozygotes. The Albert Einstein Animal Institute Committee (IACUC), using the guidelines of the NIH Office of Animal Laboratory Welfare and AAALAC, International, reviewed and approved the experiments reported here under protocol numbers 20080813, 20110803, 20140803, and 20170709 to PS. Experiments were performed in compliance with these approved protocols. Mice were sacrificed by carbon dioxide asphyxiation and cervical dislocation. Testes were dissected free of surrounding tissue and weighed. Genomic DNA was prepared from toes at 7–8 days after birth (dpp), or from tail at 10 dpp for genotyping, or extracted from liver for Southern blot analysis or PCR using a Qiagen DNeasy kit (Qiagen, Hilden, Germany). All methods were performed in accordance with the relevant guidelines and regulations approved by the Einstein IACUC. Genotyping was performed by PCR of genomic DNA using the primers shown in Supplementary Table S1, recombinant Taq polymerase, and dNTPs from New England Biolabs Inc., Ipswich, MA, United States. Quantitative PCR (qRT-PCR) to determine transgene copy number was performed as previously described (Varshney et al., 2019) using qRT-PCR primers presented in Supplementary Table S1.

### Antibodies

Antibodies (Ab) used were mouse monoclonal antibody (mAb) HA.11 clone 16B12 to detect HA (#MMS-101R, Covance, Princeton, NJ, United States), affinity-purified rabbit polyclonal antibodies (pAb) to detect ACTB (#A2066 Sigma-Aldrich, St. Louis, MO, United States); rabbit anti-human MGAT1 mAb EPR14247 (#ab180578, Abcam, Toronto, Canada); mouse mAb to GAPDH (#ab8245, Abcam); rat anti-basigin mAb clone OX114 (#B3663, LSBio, Inc., Seattle, WA, United States); horse radish peroxidase (HRP)-conjugated goat anti-rabbit IgG (#65-6120, Invitrogen Corp., Carlsbad, United States) and goat anti-mouse IgG(H + L) (#G-21040, Invitrogen); AffiniPure goat anti-rat IgG (H + L) (#112-005-003, Jackson Immunoresearch Laboratories, Inc., West Grove, PA, United States).

## MALDI-IMS

Testes of ∼90-day mice were dissected free of surrounding tissue and fixed in 10% buffered formalin. After 48 h at room temperature (RT), formalin was removed, the testis was cut vertically, each half was put into a cassette, submerged in 70% ethanol at RT and given for paraffin embedding and sectioning to the Histotechnology and Comparative Pathology Core Facility at Albert Einstein. Sections (6 μm) of formalin-fixed, paraffin-embedded (FFPE) testis were mounted on Indium-Tin-Oxide (ITO)-coated glass slides. Sections were deparaffinized in xylene three times for 5 min each, rehydrated in an ethanol/water series of 100, 95, 70% ethanol:water, and subjected to antigen retrieval in citraconic anhydride buffer at pH 3 using a steamer for 30 min (Powers et al., 2014). PNGase F Prime^TM^ (N-Zyme Scientifics LLC, Doylestown, PA, United States; 0.1 μg/μl in HPLC-grade water) was coated on each slide using an ImagePrep Station (Bruker Daltonics Inc., Bullerica, MA, United States). Slides were incubated at 37°C for 2 h in a humidified chamber and then dried in a vacuum desiccator for 20 min. A slide image was scanned using a flatbed scanner at 1,600 dpi resolution for use in selecting fiducial marks to ensure that the laser was in position. Slides were then coated with 2,5,dihydroxybenzoic acid (DHB) matrix (30 mg/ml in acetonitrile (AcN), 0.1% trifluoroacetic (TFA) acid and 0.1 mM NaCl) using the Imageprep station. N-glycan imaging was performed by scanning and acquiring spectra from the entire section (m/z 900–4,500) on an Ultraflextreme MALDI-TOF/TOF mass spectrometer equipped with a SmartBeam II laser at 1 kHz and 100 μm raster width. MALDI-TOF data were processed by FlexImaging software (v 4.0) to generate N-glycan ion maps. For MS/MS analysis, the N-glycans on the tissue surface were extracted with 25% AcN, 0.1%TFA, mixed with DHB matrix (12 mg/ml in 50% AcN, 5 mM NaOH), spotted on a MALDI target and analyzed using the Ultraflextreme MALDI-TOF/TOF mass spectrometer.

### Histology and Lectin Histochemistry

Testes from 28 or 150 dpp males were fixed in Bouin’s fixative (#100503-962, Electron Sciences, Radnor, PA, United States) for 48 h at RT and paraffin-embedded by the Einstein Histotechnology and Comparative Pathology Core Facility. Serial sections (5 μm) were collected on positively-charged slides and stained with hematoxylin and eosin (H&E). Unstained sections were used for immunohistochemistry. For lectin histochemistry, slides were microwaved in 10 mM citrate buffer (pH 6.0), incubated in 3% hydrogen peroxide in methanol (15 min, RT), and blocked in 5% goat serum (#G9023, Sigma-Aldrich, St. Louis, MO, United States) in phosphate-buffered saline (pH 7.2) containing 1 mM CaCl_2_, 1 mM MgCl_2_, 1 mM MnCl_2_ and 0.05% Tween 20 (PBST), for 60 min at RT. Sections were incubated overnight at 4°C in blocking buffer (5% goat serum in PBS containing cations) containing biotinylated lectins L-PHA (#B-1115, Vector Labs, Burlingame, CA, United States) or GSA-II (#B-1215 Vector Labs) in tris-buffered saline, pH 7.2 (TBS). After washing with PBST (no cations), sections were incubated with HRP-conjugated streptavidin (#SA-5000, Vector Labs) at 1:500 in PBST for 60 min at RT; streptavidin was detected using ABC Vectastain (#PK-6100, Vector Labs) and 3,3′diaminobenzidine (#SK-4100, Vector Labs) followed by counterstaining with hematoxylin (#MHS-16, Sigma-Aldrich). Slides were observed by light microscopy (Leica Microsystems, Wetzlar, Germany) and scanned using a Perkin Elmer P250 high capacity slide scanner (3D Histech P250 high capacity slide scanner, Perkin Elmer, Waltham, MA, United States). Specificity control was HRP-conjugated streptavidin alone.

### Western and Lectin Blot Analysis

Lysates were made from decapsulated testes of adult mice as previously described (Biswas et al., 2018). Protein was extracted in homogenization buffer (1.5% Triton X-100 in distilled water containing protease inhibitors (#05892791001, Roche Diagnostics GmbH, Mannheim, Germany) by pellet pestle (#749540000, Kontes Glass Co., Vineland, NJ, United States), centrifuged at 3,000 rpm for 10 min at 4°C, and supernatant protein was determined by Bradford assay (Bio-Rad, Hercules, CA, United States). For detection of HA and ACTB, 40–60 μg lysate protein was separated on a 10% SDS polyacrylamide gel, transferred to nitrocellulose membrane (#162-0095, Bio-Rad) in 10% methanol, blocked in 5% non-fat milk for 1 h at RT, and incubated overnight at 4°C with primary Ab. Membranes washed 3X in TBST and 1X in TBS, were then incubated with HRP-conjugated secondary antibodies in blocking solution for 1 h at RT in the dark. For detecting BSG, or GAPDH, 40–60 μg lysate protein was separated by 10% SDS-PAGE and transferred to PVDF membrane (10% methanol, 60 min), blocked in 3% fish gelatin (#G7041, Sigma-Aldrich) in TBST, and incubated in primary Abs. After washing, membranes were probed with an appropriate HRP-conjugated secondary antibody for 1 h at RT, treated with Super Signal West Pico chemiluminescent substrate (#34080, Thermo Fisher) and exposed to film. Lectin blot analysis is described in Supplementary Material.

### Statistical Analysis

All results are presented as mean ± SEM for ≥ 2 independent experiments, including replicates as noted. A one-way ANOVA or two-tailed unpaired Student’s *t*-test with Welch’s correction was used to determine *p*-values using Graph Pad Prism 7.0a (Graph Pad Software Inc., La Jolla, CA, United States) as noted.

## Results

**FIGURE 1:**
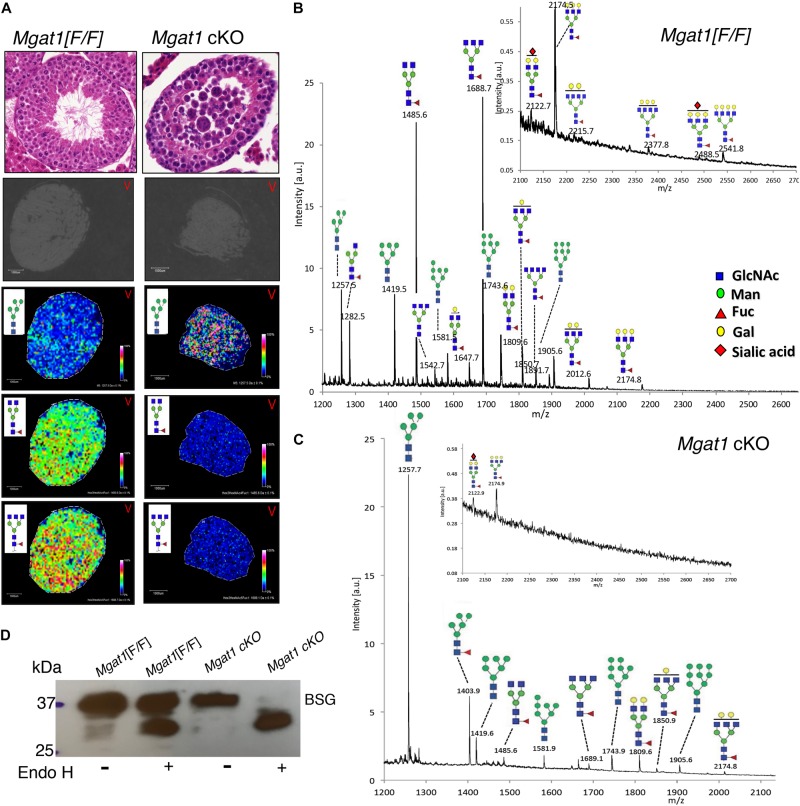
MALDI-IMS of N-glycans from *Mgat1* cKO and control testis sections. **(A)** Top panels. H&E staining of 6 μm sections from paraffin-embedded testes of *Mgat1* cKO and control (*Mgat1*[F/F]) males of 90 dpp (representative of *n* = 6; scale bar 100 μm). Lower panels: Testis sections treated with PNGase F and subjected to MALDI-IMS. The expression of a particular N-glycan is shown in each panel with its relative intensity scale (representative of two sections from each of three males). Gray sections are before treatment. **(B)** Profile of *Mgat1*[F/F] MALDI-IMS spectra (representative of two sections from three males). Inset shows expanded high m/z region. **(C)** Profile of *Mgat1* cKO MALDI-IMS spectra. Inset shows expanded high m/z region. **(D)** Western blot for basigin (BSG) after Endo H treatment of testis extracts from 90 dpp control and *Mgat1* cKO males. N-glycans are drawn with sugar symbols recommended by the Symbol Nomenclature for Glycans (Varki et al., 2015; Neelamegham et al., 2019); GlcNAc, blue square; Man, green circle; Gal, yellow circle; Fuc, red triangle; Sia, red diamond.

We have previously shown that conditional deletion of *Mgat1* in spermatogonia using *Stra8-iCre* (*Mgat1* cKO) leads to a block in spermatogenesis and infertility (Batista et al., 2012; Biswas et al., 2018). The loss of MGAT1 occurred in all testicular germ cells based on imaging of testis sections using MALDI-Imaging mass spectrometry (MALDI-IMS) (Figure 1A). The accumulation of the substrate of MGAT1 (Man_5_GlcNAc_2_Asn) and the marked reduction of complex N-glycans in the mutant is apparent in the density maps for individual N-glycans (Figure 1A) and in the MS profiles (Figures 1B,C). Consistent with this is the fact that the three complex N-glycans of basigin (BSG), a known target of MGAT1 in *Mgat1* cKO testis, were removed by endoglycosidase H (Endo H), which cleaves oligomannosyl but not complex N-glycans (Figure 1D), as shown previously (Batista et al., 2012).

**FIGURE 2:**
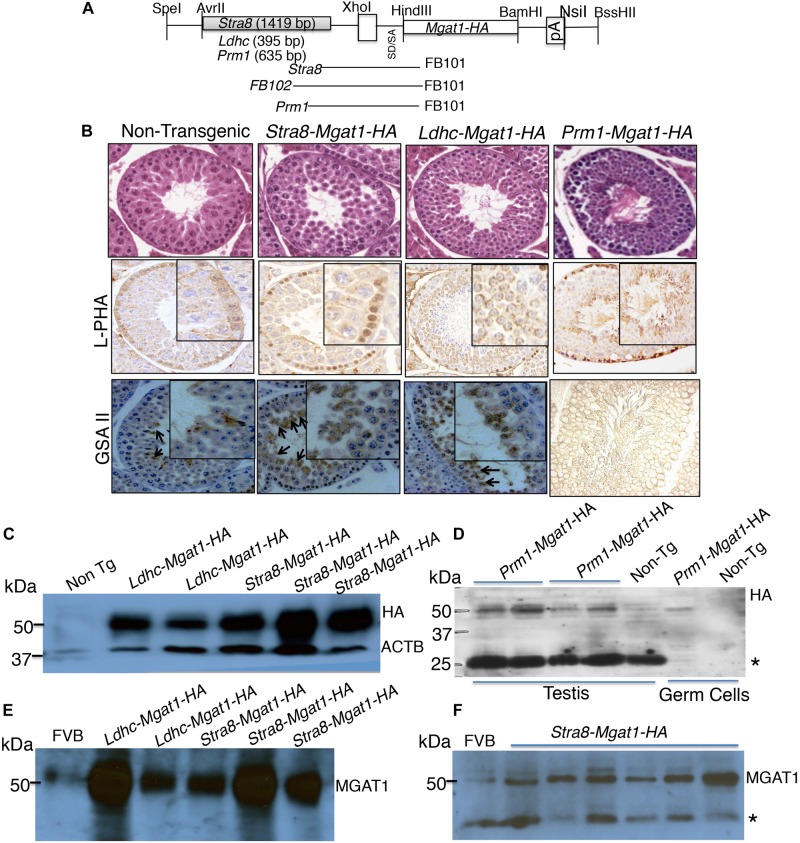
Generation and characterization of *Mgat1* transgenic mice. **(A)** Schematic of cDNA constructs used to generate transgenic mice. Expression of *Mgat1*, with a C-terminal *HA* tag (*Mgat1-HA*) was driven by germ cell specific promoters *Stra8, Ldhc* and *Prm1*, respectively. The primers for genotyping spanned the promoter and cDNA sequences so that a transgene-specific region was amplified (Supplementary Table S1). **(B)** Top panels. Representative images of H&E staining of adult non-transgenic (Control) and transgenic testis sections at 28 dpp. Scale bar 50 μm. No morphological changes were observed for *Mgat1* mis-expressed in spermatogonia, spermatocytes, or spermatids at 28 dpp (8–12 animals per transgene were analyzed; 100 tubules counted per section, two sections per mouse). Middle panels. Binding of L-PHA to testis sections from 28 dpp control males (*n* = 3, two sections per mouse), and transgenic males (*n* = 4–6, two sections per mouse). Scale bar 100 μm. Bottom panels. Binding of GSA II to testis sections from 28 dpp control (*n* = 3) and transgenic (*n* = 4–6) males. Scale bar 100 μm. **(C)** Western blot analysis of HA expression in testis extracts from control and transgenic mice with expression in spermatogonia or spermatocytes. **(D)** Western blot analysis for HA in left and right testis extracts from two *Prm1*-*Mgat1*-*HA* males and a non-transgenic male. Extracts of germ cells purified from a transgenic and a non-transgenic male are also shown. **(E,F)** Western blot analyses of *Stra8-Mgat1*-*HA* and *Ldhc-Mgat1*-*HA* transgenic testis lysates for expression of MGAT1 compared to FVB controls. The ACTB control for **(C)** is the same for **(E)**. *Non-specific loading control.

To determine the germ cell type that requires complex N-glycans for survival or differentiation, we used a transgenic rescue strategy. A *Mgat1-HA* cDNA was cloned under the control of the *Stra8* promoter from *Stra8-iCre* (Sadate-Ngatchou et al., 2008), or the *Ldhc* promoter from *Ldhc-LacZ* (Li et al., 1998; Goldberg et al., 2010), or the *Prm1* promoter from *Prm1-Cre* (O’Gorman et al., 1997; Figure 2A). Several transgenic founders were obtained following injection of each cDNA into fertilized eggs (5 for *Stra8-Mgat1-HA*, 4 for *Ldhc-Mgat1-HA*, and 16 for *Prm1-Mgat1-HA*). However, each transgene was transmitted through the germline by only one founder, respectively. Transgene expression was characterized by histology, lectin histochemistry and western blot analysis for HA and MGAT1. Testis sections from each transgenic strain stained with H&E exhibited no differences in spermatogenesis from non-transgenic control sections (Figure 2B). Lectin blot analysis revealed increased signal for the plant lectins phytohemagglutinin-L (L-PHA) and *Griffonia simplicifolia* II (GSA II) in sections from mice expressing each transgene compared to non-transgenic controls (Figure 2B). Both lectins bind to complex N-glycans and binding was increased as expected due to expression of the *Mgat1-HA* transgene. Western blot analysis for expression of HA or MGAT1 in testis lysates showed that the signal for each was markedly increased in *Stra8-Mgat1-HA* and *Ldhc-Mgat1-HA* transgenic males (Figures 2C,E,F) compared to non-transgenic littermates or wild type males. The HA signal of the *Prm1-Mgat1-HA* transgene was also increased in both testis and germ cell lysates (Figure 2D). The *Prm1-Mgat1-HA* strain was investigated in additional detail. Southern analysis showed that the transgene was inserted as a linear concatemer (Supplementary Figure S1). Quantitative PCR of genomic DNA from liver revealed that the *Prm1* promoter and the *Mgat1* gene were present in ∼8–11 copies in the three mice analyzed, compared to two copies of each in non-transgenic liver DNA (Supplementary Material). Lectin blot analysis showed a large increase in the binding of L-PHA to glycoproteins in *Prm1-Mgat1-HA* testes, consistent with increased expression of complex N-glycans due to the transgene (Supplementary Figure S2). By contrast, binding of *Galanthus nivalis* lectin (GNA) which recognizes oligomannosyl N-glycans was very low in proteins from transgenic testes, again consistent with high conversion of oligomannosyl to complex N-glycans by MGAT1. Proteins from *Mgat1* cKO testes or Lec1 CHO cells that lack complex N-glycans also bound GNA well, but L-PHA poorly (Chen and Stanley, 2003; Huang and Stanley, 2010). Non-transgenic testis extract and wild type CHO cells bound L-PHA well, and GNA poorly.

The fertility of each of the transgenic strains was similar to non-transgenic littermates and each transgene was transmitted ∼50% of the time. Thus, there were no apparent deleterious consequences of mis-expressing or over-expressing *Mgat1-HA* in spermatogonia (*Stra8*), spermatocytes (*Ldhc*), or spermatids (*Prm1*).

**FIGURE 3:**
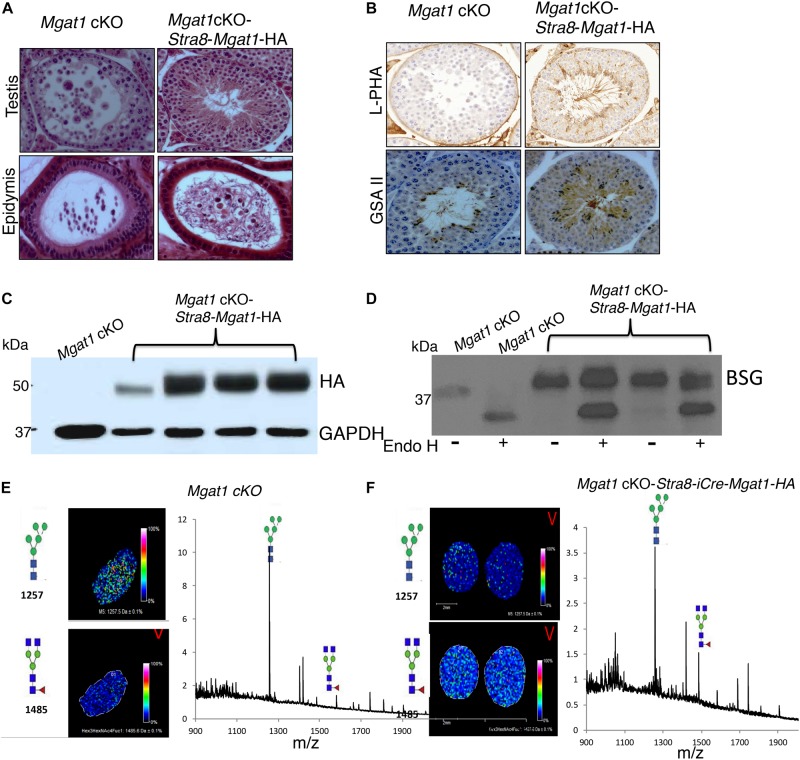
Rescue of *Mgat1* cKO males by the *Stra8-Mgat1-HA* transgene. **(A)** H&E staining of testes and epididymi from *Mgat1* cKO-*Stra8-Mgat1-HA* males of 150 dpp (one testis, duplicate sections, eight males). In *Mgat1* cKO testis MNCs are apparent. In *Mgat1* cKO-*Stra8-Mgat1-HA* testes MNCs were rare. In both *Mgat1* cKO and *Mgat1* cKO-*Stra8-Mgat1-HA* rescue mice, cell debris was present in the epididymal lumen. Scale bar 100 μm. **(B)** Lectin staining with L-PHA and GSA II in *Mgat1* cKO compared to *Mgat1* cKO-*Stra8-Mgat1-HA* testis shows that the transgene rescued lectin binding to complex N-glycans (*n* = 6, two sections per mouse). Scale bar 100 μm. **(C)** Western blot analysis for HA and ACTB in testes of non-Tg control (*n* = 3), six rescue mice from *Mgat1* cKO-*Stra8-Mgat1-HA* and 6 from *Mgat1* cKO*-Ldhc-Mgat1-HA* for expression of the transgene. **(D)** Western analysis for BSG in testicular germ cells from non-Tg (*n* = 3) and *Mgat1* cKO-*Stra8-Mgat1-HA* males (*n* = 3) with and without digestion by Endo H. **(E,F)** MALDI-IMS of testis sections from *Mgat1* cKO control and *Mgat1* cKO-*Stra8-Mgat1-HA* rescue mice showing increased expression of complex N-glycans in the rescue mouse. Glycan symbols are described in the legend to Figure 1.

To investigate whether the respective transgenes could rescue spermatogenesis in a *Mgat1* cKO male, each transgene was crossed onto *Mgat1*[F/F] males, which were then crossed with *Mgat1*[F/F]:*Stra8-iCre* females, to generate *Mgat1* cKO-*Stra8-Mgat1-HA*, *Mgat1* cKO-*Ldhc-Mgat1-HA* or *Mgat1* cKO-*Prm1*-*Mgat1-HA* “rescue” males, respectively. Several “rescue” males for each transgene were mated to wild type FVB females and the progeny were genotyped to determine whether *Mgat1* had been deleted by *Stra8-iCre*, and to show that the respective *Mgat1-HA* transgene was present. Six *Mgat1* cKO males expressing the *Stra8*-*Mgat1-HA* transgene were fertile and gave an average of 7 pups per litter from 10 litters. Histological analysis revealed ∼13% testis tubules contained MNCs and ∼3% epididymi contained cellular debris amongst their sperm (Figure 3A). However, the majority of testis tubules were rescued. Thus, lectin histochemistry showed that complex N-glycans bound by the lectins L-PHA and GSA II were present in *Mgat1* cKO-*Stra8-iCre-Mgat1-HA* transgenic mice, but absent from *Mgat1* cKO control (Figure 3B). Western analysis showed the expression of the transgene was robust (Figure 3C) and that BSG N-glycans were ∼70% resistant to Endo H digestion, consistent with the presence of mainly complex N-glycans (Figure 3D). MALDI-IMS of testis sections showed a marked increase in biantennary complex N-glycans in *Mgat1* cKO-*Stra8-Mgat1-HA* males compared to *Mgat1* cKO males (Figures 3E,F). The latter exhibited the characteristic accumulation of MGAT1 substrate. Thus, the *Stra8*-driven transgene substantially rescued the deletion of *Mgat1* in the majority of testis tubules leading to apparently normal fertility.

**FIGURE 4:**
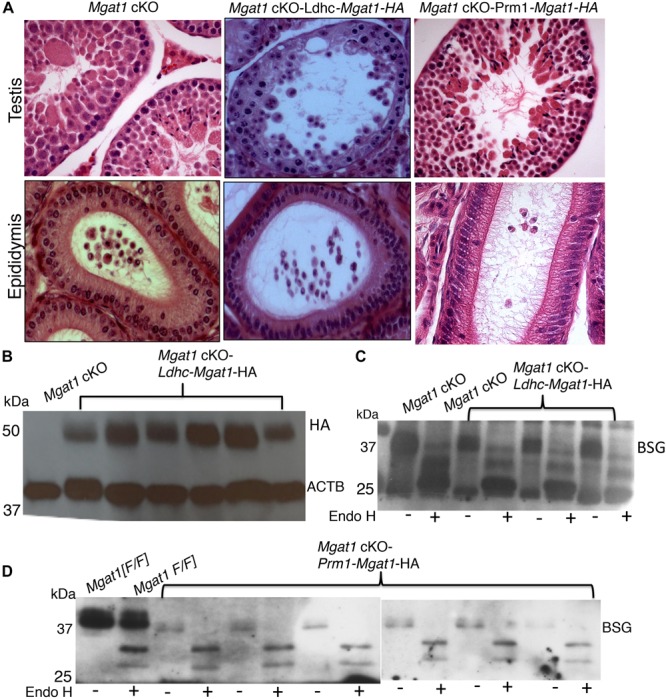
Lack of rescue by an *Mgat1-HA* transgene expressed in spermatocytes or spermatids of *Mgat1* cKO males. **(A)** Representative H&E images of testis sections and epididymi from *Mgat1* cKO mice (*n* = 6, one testis, duplicate sections), *Mgat1* cKO-*Ldhc-Mgat1-HA* (*n* = 10), and *Mgat1* cKO-*Prm1-Mgat1-HA* males (*n* = 6) at 150 dpp. Damaged tubules with MNCs are seen in *Mgat1* cKO as expected, as well as in *Ldhc-Mgat1-HA* (*n* = 10) and *Prm1-Mgat1-HA* (*n* = 6) testis sections. Epididymal lumen lacks spermatozoa and cell debris is present (scale bar 100 μm). **(B)** Western blot analysis for HA in *Mgat1* cKO-*Ldhc-Mgat1-HA* (*n* = 6) compared to non-transgenic testes. **(C)** Western blot analysis for BSG in *Mgat1* cKO and *Mgat1* cKO-*Ldhc-Mgat1-HA* testis extracts incubated with or without Endo H (*n* = 3 males). **(D)** Testis extracts from *Mgat1* cKO and *Mgat1* cKO-*Prm1-Mgat1-HA* incubated with or without Endo H were subjected to Western blotting for BSG (*n* = 6). Two gels were combined.

By contrast, 10 *Mgat1* cKO-*Ldhc-Mgat1-HA* transgenic males and 4 of 6 *Mgat1* cKO-*Prm1*-*Mgat1-HA* males gave no progeny when housed with wild type females, several of which were known to be fertile. This was despite robust expression of the *Mgat1-HA* transgene in each strain (Figures 4A,B). Males that gave no progeny exhibited the typical disruption of spermatogenesis of *Mgat1* cKO males (Batista et al., 2012; Biswas et al., 2018) with many MNCs per tubule, no sperm and debris in the epididymis (Figure 4A and Supplementary Figure S3). The N-glycans of BSG were fully sensitive to removal by Endo H in these males (Figures 4C,D), showing there were no complex N-glycans on BSG. The two *Prm1-Mgat1-HA* males that did not exhibit 100% deletion of the *Mgat1* gene, had slightly rescued spermatogenesis and were fertile (Supplementary Figure S3), apparently due to MGAT1 activity derived from the *Mgat1[F]* alleles observed in progeny, and not to rescue by the *Prm1-Mgat1-HA* transgene. In the final analysis, only the *Stra8*-*Mgat1-HA* transgene rescued the fertility of *Mgat1* cKO males.

## Discussion

Spermatogenesis requires the synthesis of several glycan species, including complex N-glycans (Batista et al., 2012; Biswas et al., 2018; Akintayo and Stanley, 2019). However, determining mechanistic bases for these requirements is a challenge since glycans are expressed on many different glycoproteins and glycolipids. We have taken a combined genetic/molecular/biochemical approach to identify features of the requirement for complex N-glycans during spermatogenesis. We showed that deletion of *Mgat1* at ∼3 dpp in male germ cells manifests morphologically at about 26 dpp as fusion of spermatids (Biswas et al., 2018). However, molecular changes can be observed earlier. Microarray analyses of transcripts from male germ cells of 22 or 23 dpp mice revealed gene expression differences consistent with the premature activation of spermatogenesis and spermiogenesis genes in *Mgat1* cKO germ cells, and implicated reduced EGF/PDGF receptor signaling. Indeed, there was a marked reduction in pERK1/2 in germ cells of *Mgat1* cKO males compared to controls. In this paper, we continue the investigation into roles for complex N-glycans in spermatogenesis by asking in which germ cells MGAT1 is required for spermatogenesis to proceed normally.

To address this question, we engineered transgenic mice expressing a *Mgat1-HA* cDNA in spermatogonia, or spermatocytes, or spermatids with the aim of determining which, if any, could rescue the defects in *Mgat1* cKO spermatogenesis. First, we investigated whether mis-expression of *Mgat1-HA* in specific germ cells would disrupt their function. We found that spermatogenesis and fertility were similar to non-transgenic controls in each transgenic strain. That is, we demonstrated that mis- or over-expression of *Mgat1* in spermatogonia or spermatocytes or spermatids did not apparently affect spermatogenesis, and did not significantly alter fertility. Thus, we could investigate whether one or more of the transgenes would rescue the spermatogenic defects in *Mgat1* cKO mice. We report here that the only transgene to rescue *Mgat1* cKO males was the *Mgat1-HA* expressed in spermatogonia under the *Stra8* promoter. The degree of rescue was high but not complete, as shown by the presence of ∼13% testicular tubules that contained MNCs, epidydimi with pyknotic cells in the lumen, the presence of an increased amount of Man_5_GlcNAc_2_Asn in germ cells by MALDI-IMS, and the fact that the N-glycans of BSG had an increased proportion of oligomannosyl N-glycans sensitive to Endo H. Nevertheless, *Mgat1* cKO-*Stra8-Mgat1-HA* males were as fertile as controls. By contrast, expression of *Mgat1-HA* in spermatocytes under the *Ldhc* promoter, or spermatids under the *Prm1* promoter, did not rescue the *Mgat1* cKO phenotype. These findings are striking and point to spermatogonia as being the key germ cell in which complex N-glycans must function for spermatogenesis to proceed. It is interesting that the MNCs observed in the absence of MGAT1 are formed by round and elongated spermatids (Batista et al., 2012; Biswas et al., 2018). Spermatogonia and spermatocytes appear normal, and are present in their usual numbers, when *Mgat1* is deleted in spermatogonia (Biswas et al., 2018). However, the increased level of apoptosis present in *Mgat1* cKO germ cells (Batista et al., 2012) probably affects primarily spermatocytes. In addition, the molecular changes observed at 22 or 23 days after birth (Biswas et al., 2018), largely before spermatids are present. presumably lead to the dramatic fusion of spermatids that begins at ∼26 days after birth.

Based on the importance of MGAT1 in mouse spermatogenesis and fertility (Batista et al., 2012; Biswas et al., 2018), it is to be expected that mutations in the *MGAT1* gene are a source of infertility in men. However, a recent comprehensive review of mutations associated with infertility in men did not report any with a mutation in the *MGAT1* gene (Oud et al., 2019). Nevertheless, our studies suggest that *MGAT1* is a strong candidate and will likely be revealed as a basis for infertility in future genomic studies. MGAT1 is also a potentially excellent target for a male contraceptive strategy. A small molecule inhibitor or a peptide (Huang and Stanley, 2010) that inhibits MGAT1 activity would ostensibly have few effects on spermatogonia or spermatocytes (Biswas et al., 2018), but would induce fusion of spermatids and prevent the formation of sperm (Batista et al., 2012), thus having contraceptive activity. Moreover, these effects could be reversible, since spermatogonia are not observably impacted by inhibition of MGAT1 activity (Biswas et al., 2018).

## Data Availability Statement

All datasets generated for this study are included in the article/Supplementary Material.

## Ethics Statement

Animal experiments were reviewed and approved by the Einstein Institutional Animal Care and Use Committee under protocol numbers 20110803, 20080813, 20140803, and 20170709.

## Author Contributions

BB generated transgenic and rescue mice, characterized them by histology, lectin histochemistry, western blotting, and fertility and co-wrote the manuscript. FB generated and characterized the *Prm1* transgenic and rescue lines and read the manuscript. AA bred experimental mice, characterized rescue mice, and edited the manuscript. JA performed and interpreted MALDI-IMS and edited the manuscript. PS conceived and guided the experiments, acquired funding, analyzed data, and co-wrote the manuscript.

## Conflict of Interest

The authors declare that the research was conducted in the absence of any commercial or financial relationships that could be construed as a potential conflict of interest.
