# Supplementary material for: Transgenic Rescue of Spermatogenesis in Males With Mgat1 Deleted in Germ Cells
Source: Front Cell Dev Biol. 2020 Apr 2;8:212. doi: 10.3389/fcell.2020.00212 (PMC7142241; doi:10.3389/fcell.2020.00212)
Supplement: Supplementary file 1 [file Data_Sheet_1.pdf]

Supplementary Material

Transgenic Rescue of Spermatogenesis in Males with *Mgat1* Deleted in Germ Cells

Barnali Biswas[1#], Frank Batista[1], Ayodele Akintayo[1#], Jennifer Aguilan[2] and Pamela Stanley[1,*]

[1] Departments of Cell Biology, Albert Einstein College of Medicine, New York, NY, United States, [2] Laboratory for Macromolecular Analysis and Proteomics, Department of Pathology, Albert Einstein College of Medicine, New York, NY, United States.

[#] **Present address:**

Barnali Biswas, Dept of Innate Immunity, ICMR-NIRRH, J M Street, Parel, Mumbai-12, India

Ayodele Akintayo, ZFMK, Leibniz Institute for Animal Biodiversity, Bonn, Germany

***Correspondence:**

Pamela Stanley pamela.stanley@einsteinmed.org; pamela.stanley@einstein.yu.edu

Supplementary Methods

Southern blot analysis.

Genomic DNA was extracted from liver of *Prm1-Mgat1-HA* males and non-transgenic littermates using a Qiagen DNeasy kit (Qiagen, Hilden, Germany), digested with BglII or PvuII, and subjected to agarose gel electrophoresis. After transfer to membrane, blots were hybridized to a P^{32}-labeled probe specific for either the *Prm1* or *Mgat1* gene, respectively, as previously described (Zhang et al., 1999).

Lectin blot analysis

Testis extracts from *Prm1-Mgat1-HA* males were prepared as described (Biswas et al., 2018), 60 µg protein per sample was subjected to SDS-PAGE, the gel was transferred to Polyscreen™ polyvinylidene difluoride (PVDF) membrane and probed with biotinylated lectins L-PHA or

GNA (5 μg/ml, Vector Laboratories, Burlingame, CA), followed by detection with Streptavidin-horse radish peroxidase, as previously described (Song et al., 2010).

REFERENCES

Batista, F., Lu, L., Williams, S.A., and Stanley, P. (2012). Complex N-Glycans Are Essential, but Core 1 and 2 Mucin O-Glycans, O-Fucose Glycans, and NOTCH1 Are Dispensable, for Mammalian Spermatogenesis. *Biol Reprod* 86**,** 179, 171-112.

Biswas, B., Batista, F., Sundaram, S., and Stanley, P. (2018). MGAT1 and Complex N-Glycans Regulate ERK Signaling During Spermatogenesis. *Sci Rep* 8**,** 2022.

Chen, W., and Stanley, P. (2003). Five Lec1 CHO cell mutants have distinct Mgat1 gene mutations that encode truncated N-acetylglucosaminyltransferase I. *Glycobiology* 13**,** 43-50.

Neelamegham, S., Aoki-Kinoshita, K., Bolton, E., Frank, M., Lisacek, F., Lutteke, T., O'boyle, N., Packer, N.H., Stanley, P., Toukach, P., Varki, A., Woods, R.J., and Group, S.D. (2019). Updates to the Symbol Nomenclature for Glycans guidelines. *Glycobiology* 29**,** 620-624.

Song, Y., Aglipay, J.A., Bernstein, J.D., Goswami, S., and Stanley, P. (2010). The bisecting GlcNAc on N-glycans inhibits growth factor signaling and retards mammary tumor progression. *Cancer Res* 70**,** 3361-3371.

Varki, A., Cummings, R.D., Aebi, M., Packer, N.H., Seeberger, P.H., Esko, J.D., Stanley, P., Hart, G., Darvill, A., Kinoshita, T., Prestegard, J.J., Schnaar, R.L., Freeze, H.H., Marth, J.D., Bertozzi, C.R., Etzler, M.E., Frank, M., Vliegenthart, J.F., Lutteke, T., Perez, S., Bolton, E., Rudd, P., Paulson, J., Kanehisa, M., Toukach, P., Aoki-Kinoshita, K.F., Dell, A., Narimatsu, H., York, W., Taniguchi, N., and Kornfeld, S. (2015). Symbol Nomenclature for Graphical Representations of Glycans. *Glycobiology* 25**,** 1323-1324.

Zhang, A., Potvin, B., Zaiman, A., Chen, W., Kumar, R., Phillips, L., and Stanley, P. (1999). The gain-of-function Chinese hamster ovary mutant LEC11B expresses one of two Chinese hamster FUT6 genes due to the loss of a negative regulatory factor. *J Biol Chem* 274**,** 10439-10450.

Figure Legends

Supplementary Figure 1. Southern blot analysis of the *Prm1-Mgat1-HA* transgene. A diagram of the *Prm1-Mgat1-HA* transgene with relevant restriction sites is shown. Both probes detected the respective endogenous gene (asterisks) and the *Prm1-Mgat1-HA* transgene. The transgene was overexpressed compared to each endogenous gene and, based on the size of predicted gene products, was incorporated into the genome in a linear concatemer.

Supplementary Figure 2. Lectin blot analysis of *Prm1-Mgat1-HA* transgenic male testes. L-PHA binds to complex N-glycans as shown in the Chinese hamster ovary (CHO) cell extract. The *Mgat1*[F/F] testis extract had little protein as shown by the low signal for ACTB. L-PHA does not bind to glycoproteins lacking complex N-glycans, as shown by the low signal for *Mgat1* cKO extract and the Lec1 CHO mutant extract. Lec1 cells lack MGAT1 activity (Chen and

Stanley, 2003). GNA binds to oligomannosyl N-glycans that predominate in cells lacking MGAT1. GNA binds poorly to cells expressing predominantly complex N-glycans. *Prm1-Mgat1-HA* male testis expresses much greater levels of complex N-glycans and thus MGAT1, compared to non-transgenic testis, or CHO cells expressing endogenous levels of MGAT1. Predominant complex and oligomannosyl N-glycans are shown over the different control samples. Symbols for sugars are based on the Symbol nomenclature for N-glycans (Varki et al., 2015;Neelamegham et al., 2019). GlcNAc, blue square; Man, green circle; Gal, yellow circle; Fuc, red triangle; Sia, red diamond.

Supplementary Figure 3. Histological analysis of testes from *Prm1-Mgat1-HA* males. Six transgenic male testes were housed with 1-2 females for 3 months. Two males had progeny as noted, and 4 males had no progeny. After 3 months, the males were sacrificed, testes dissected, fixed in Bouin's, sectioned (5 μm) and stained with hematoxylin and eosin. Most, if not all, of the testis tubules in each transgenic male contained multinucleated cells (MNC). However, PMG75 and PMG82 had fewer MNCs, both had progeny, and both exhibited inefficient deletion of *Mgat1* floxed alleles. PMG75 transmitted a deleted allele to 38% of pups and PMG82 to 66%. Since *Mgat1[F/+]:Stra8-iCre* heterozygotes are as fertile as wild type (Batista et al., 2012), it is probable that the fertility of the 2 fertile transgenic males is due to undeleted *Mgat1*[F] allele(s) observed in progeny, and not to rescue by the *Prm1-Mgat1-HA* transgene. However, histological analysis of 100 tubules from an infertile male revealed 14% unaffected tubules suggesting that some rescue had occurred.

Table S1. Primer sequences for genotyping (Gene) or qPCR

Gene Name	Primers	Assay	Primer Sequence	Product Length (bp)
Mgat1[F/+]	PS585-Fw<br>PS586-Rv	Gene	TGCAAGCCAACACTTGTCTC<br>GAGACCTGCTTACTGCAGCC	561 [F]<br>421 [+]
Mgat1[-]	BB3-Fw	Gene	CTGCTCCAGGACAAGAGCCA	560 [-]
	BB4-Rv		GAGACCTGCTTACTGCAGCC	
Stra8-iCre	Stra-Fw	Gene	AGATGCCAGGACATCAGGAACCTG	380
	Stra-Rv		ATCAGCCACACCAGACACAGAGATC	
Stra8-Mgat1-HA	Stra8-Fw<br>Mgat1-Rv	Gene	GACAGGGCTGTGATTGGTTC<br>ATGATAGCACCCCAAAGCAC	402
Ldhc-Mgat1-HA	Ldhc-Fw<br>Mgat1-Rv	Gene	GTCTACAGAGTTCCAGGACG<br>ATGATAGCACCCCAAAGCAC	672
Prm1-Mgat1-HA	Prm1-Fw<br>Mgat1-Rv	Gene	AAGCAGGTGTGTGGCACTTA<br>ATGATAGCACCCCAAAGCAC	450
Prm1	FB113-Fw	qPCR	TCCAAACACTGCTCTGCATC	180
	FB114-Rv		GAGAATGCTGCTGAGGGAAC	
Mgat1	FB115-Fw	qPCR	CTTCACCCAGTTGGACCTGT	168
	FB116-Rv		GCCTTGAAGCTGTCTCTGCT	
Alb	Alb-Fw	qPCR	CAGGTGTCAACCCCAACTCT	100
	Alb-Rv		CCACACAAGGCAGTCTCTGA	

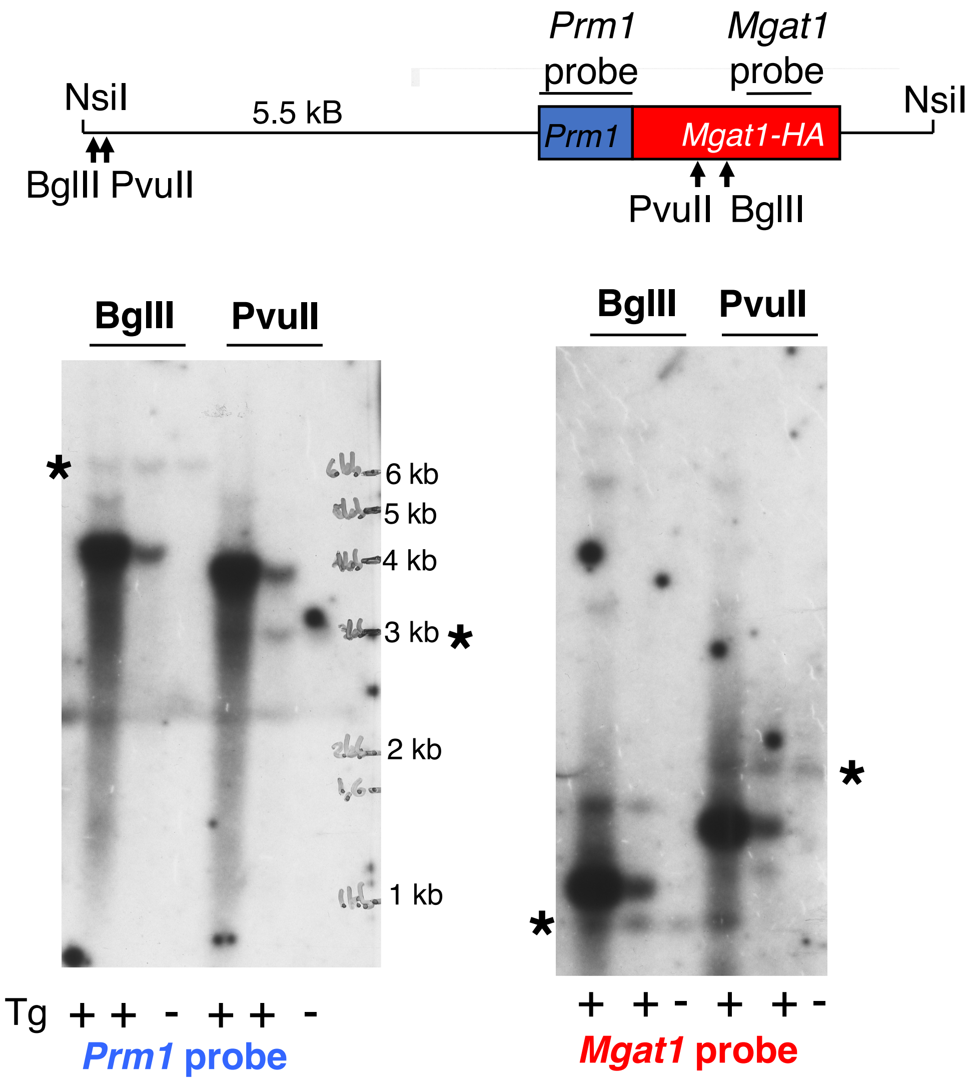
Prm1 probe
Mgat1 probe
NsiI
5.5 kB
Prm1
Mgat1-HA
NsiI
BglII PvuII
PvuII BglII
BglII
PvuII
*
6 kb
5 kb
4 kb
3 kb
*
2 kb
1 kb
Tg + + - + + -
Prm1 probe
BglII
PvuII
*
*
+ + - + + -
Mgat1 probe

Fig. S1

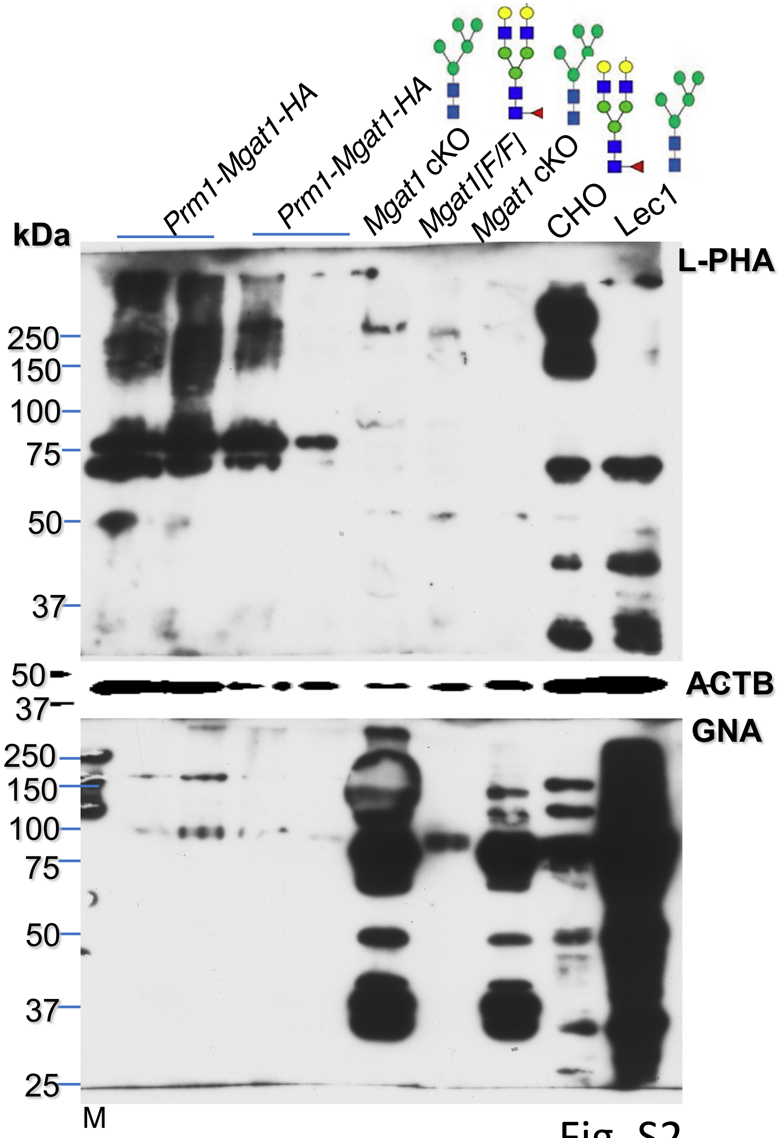

Prrm1-Mgat1-HA
Prrm1-Mgat1-HA
Mgat1 cKO
Mgat1[F/F]
Mgat1 cKO
CHO
Lec1
kDa
L-PHA
250
150
100
75
50
37
50
37
ACTB
GNA
250
150
100
75
50
37
25
M

Fig. S2

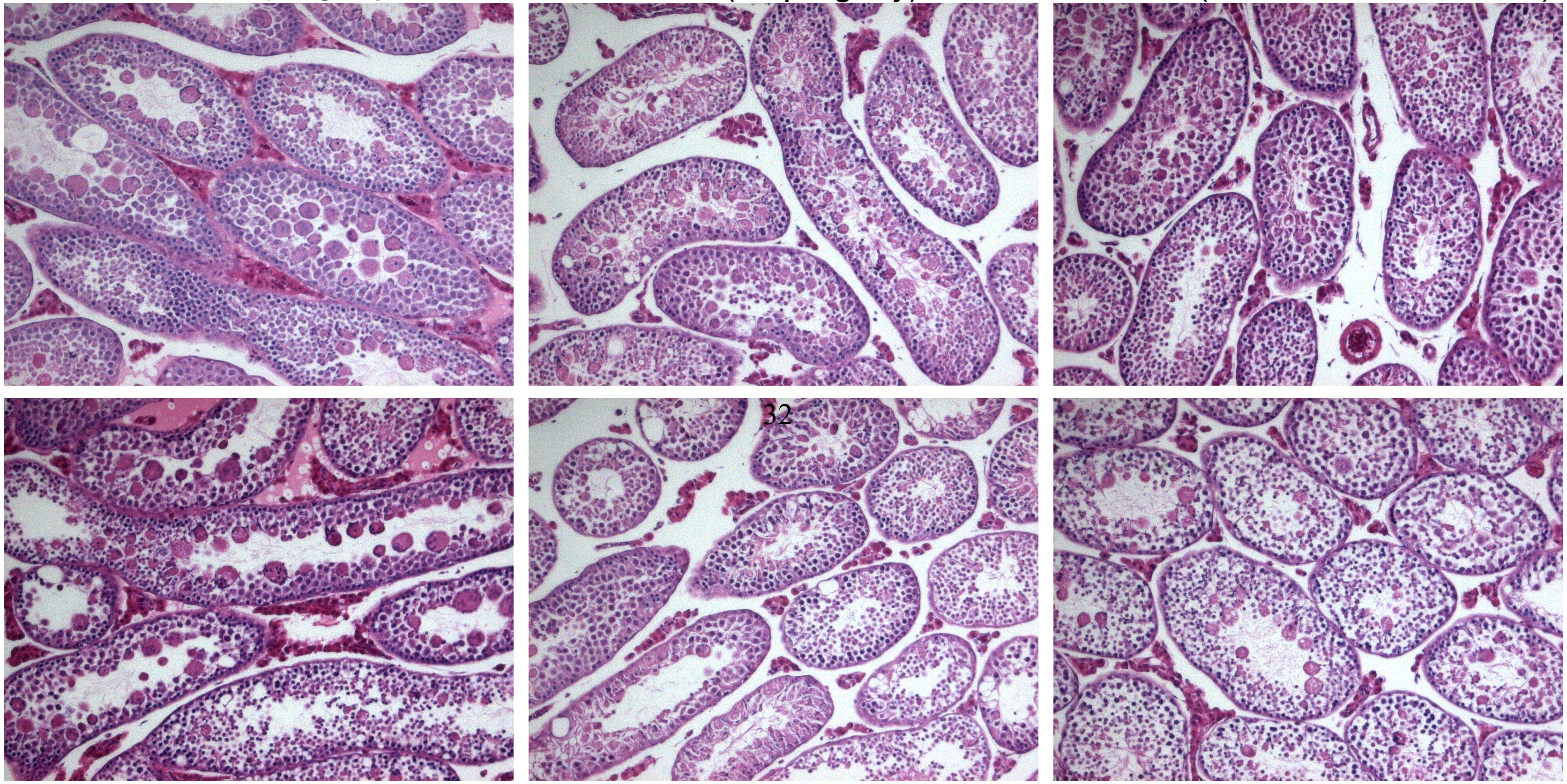
PMG70 (no progeny)
PMG71 (no progeny)
PMG75 (38% deletion, 2 litters)
PMG76 (no progeny)
PMG82 (66% deletion, 1 litter)
PMG85 (no progeny)

Fig. S3
